# A Novel Herbal Medicine KIOM-MA Exerts an Anti-Inflammatory Effect in LPS-Stimulated RAW 264.7 Macrophage Cells

**DOI:** 10.1155/2012/462383

**Published:** 2012-11-27

**Authors:** You-Chang Oh, Won-Kyung Cho, Yun Hee Jeong, Ga Young Im, Aeyung Kim, Youn-Hwan Hwang, Taesoo Kim, Kwang Hoon Song, Jin Yeul Ma

**Affiliations:** Korean Medicine (KM)-Based Herbal Drug Research Group, Korea Institute of Oriental Medicine, 461-24 Jeonmin-Dong, Yuseong, Daejeon 305-811, Republic of Korea

## Abstract

KIOM-MA was recently reported as a novel herbal medicine effective for atopic dermatitis and asthma. In this study, we have demonstrated the inhibitory effect of KIOM-MA on proinflammatory mediator produced in lipopolysaccharide (LPS)-stimulated RAW 264.7 cells. KIOM-MA significantly inhibited the expression of inducible nitric oxide synthase (iNOS) and cyclooxygenase-2 (COX-2) as well as nitric oxide (NO) and prostaglandin E_2_ (PGE_2_). Consistent with the inhibitory effect on PGE_2_, KIOM-MA suppresses the LPS-induced migration of macrophages and gelatinase activity and the expression of matrix metalloprotease-9 (MMP-9) in a dose-dependent manner. Additionally, KIOM-MA showed a strong suppressive effect on the inflammatory cytokines production such as tumor necrosis factor-**α** (TNF-**α**) and interleukin-6 (IL-6). We also found that KIOM-MA inhibits the activation of nuclear factor-**κ**B (NF-**κ**B) and represses the activity of extracellular signal-regulated kinase (ERK), p38, and c-Jun NH_2_-terminal kinase (JNK) mitogen-activated protein kinases (MAPKs). Taken together, we elucidated the mechanism of anti-inflammatory effect of KIOM-MA using RAW 264.7 cells stimulated by LPS.

## 1. Introduction

Inflammation is an initial host immune response to protect body from infection or tissue injury. In normal state, inflammation-related mediators, such as NO, PGE_2_, and cytokines, generated from immune cells including macrophage cells take an essential role in host survival and tissue repair [1]. However, these inflammatory mediators are overexpressed by stimuli and could cause autoimmune diseases and inflammatory disorders [2–4]. Especially, NO produced by iNOS and PGE_2_ synthesized by COX-2 is well-known mediators, responsible for inflammatory diseases. In addition, PGE_2_ produced by LPS stimulation takes a role in the migration of macrophage cells to the inflammatory sites [5] and induces MMP-9 activity and expression [6]. In addition, cytokines such as TNF-*α*, IL-1*β*, and IL-6 participate in the process of inflammatory disorders.

During inflammation, the expressions of these cytokines and their genes are regulated by NF-*κ*B, which are critically involved in the pathogenesis of various chronic inflammatory diseases [7]. In unstimulated state, NF-*κ*B is present as an inactive form, bound with I*κ*B*α* in the cytosol. When cells are stimulated by LPS, activated I*κ*B*α* kinase phosphorylates I*κ*B*α* and phosphorylated I*κ*B*α* are ubiquitinated and degraded through proteasome pathway [8]. Free NF-*κ*B is translocated into the nuclei and binds to the promoters of diverse proinflammatory mediators including COX-2, iNOS, TNF-*α*, and IL-6, ultimately resulting in gene expression. Most anti-inflammatory drugs are designed to suppress the expression of these genes by inhibiting NF-*κ*B signaling pathway [9].

Furthermore, MAPK activated by phosphorylation is well known to induce NF-*κ*B activation and to increase iNOS gene expression. Several studies have showed that specific MAPK inhibitors could downregulate iNOS gene expression [10–12].

A lot of reports demonstrated that natural herbal medicines have potential anti-inflammatory activities [13, 14]. Our group has developed novel herbal medicine KIOM-MA and recently demonstrated that KIOM-MA exhibits antiatopic dermatitis and asthma activities through inhibiting inflammatory factors [15, 16]. In this study, we evaluated anti-inflammatory effect of KIOM-MA using RAW 264.7 macrophage cells stimulated with LPS. Furthermore, we investigated the underlying mechanism of anti-inflammatory effect of KIOM-MA.

## 2. Materials and Methods

### 2.1. Materials and Reagent

RPMI 1640, fetal bovine serum (FBS), and antibiotics were purchased from HyClone (Logan, UT, USA). LPS, bovine serum albumin (BSA), 3-(4,5-dimethylthiazol-2-yl)-2,5-diphenylthiazolium bromide (MTT), and dexamethasone were purchased from Sigma (St. Louis, MO, USA). TNF-*α* and IL-6 ELISA sets were purchased from eBioscience (San Diego, CA, USA). iNOS, COX-2, NF-*κ*B p65, phospho-I*κ*B*α*, I*κ*B*α*, *β*-actin, TATA box-binding protein (TBP), phospho-ERK, ERK, phospho-p38, p38, phospho-JNK and JNK primary antibodies, and horseradish-peroxidase- (HRP-) conjugated secondary antibodies were obtained from cell signaling technology, Inc. (Boston, MA, USA). RNA extraction kit was obtained from iNtRON biotechnology (Sungnam, Republic of Korea). The oligonucleotide primers were synthesized from Bioneer (Daejeon, Republic of Korea). Liquiritin, nodakenin, icariin, glycyrrhizin, decursin, and decursinol angelate were purchased from Sigma (St. Louis, MO, USA). High-performance-liquid-chromatography- (HPLC-) grade water, acetonitrile, and glacial acetic acid were purchased from J. T. Baker (Austin, TX, USA).

### 2.2. Preparation of KIOM-MA

A novel herbal medicine, KIOM-MA, is composed of Glycyrrhizae Radix, Polygoni Cuspidati Rhizoma, Sophorae Radix, Cnidii Rhizoma, Arctii Fructus, and so forth, which were purchased from Yeongcheon Oriental Herbal Market (Yeongcheon, Republic of Korea). All voucher specimens were deposited in the herbal bank and 1840.0 g of the KIOM-MA formula were placed in 18.4 L of distilled water and then extracted by heating for 3 hours at 115°C (Gyeongseo Extractor Cosmos-600, Incheon, Republic of Korea). After extraction, the KIOM-MA was filtered out using standard testing sieves (150 *μ*m) (Retsch, Haan, Germany). The freeze-dried extract was then dissolved in phosphate buffered saline (PBS) and filtered (pore size, 0.45 *μ*m), lyophilized, and kept at 4°C prior to use.

### 2.3. Cell Culture

 RAW 264.7 cells were obtained from Korea Cell Line Bank (Seoul, Republic of Korea) and grown in RPMI 1640 medium added 10% FBS and 100 U/mL of antibiotic sulfate. The cells were incubated in a humidified 5% CO_2_ atmosphere at 37°C. To stimulate cells, the medium was changed with fresh RPMI 1640 medium and LPS (200 ng/mL) was added in the presence or absence of KIOM-MA (10, 100, 250, and 500 *μ*g/mL) for the indicated periods.

### 2.4. Cell Viability Assay

 Cytotoxicity of KIOM-MA was analyzed using an MTT assay. Cells were seeded at a density of 5 × 10^4^ cells/mL in 96-well culture plates. LPS or KIOM-MA was added to the cells and incubated for 48 hours at 37°C with 5% CO_2_. MTT solutions (5 mg/mL in a PBS) were added to each well and the cells were incubated for another 4 hours. The supernatant was discarded and then the formazan was dissolved in dimethyl sulfoxide (DMSO). The optical density was read at 570 nm.

### 2.5. Measurement of NO Production

NO production was analyzed by measuring nitrite in the supernatants of cultured RAW 264.7 macrophage cells. The cells were cultured in 96-well culture plates (5 × 10^5^ cells/mL) and pretreated with KIOM-MA before LPS stimulation. The supernatant was mixed with the Griess reagent and incubated at room temperature (RT) for 5 min [13]. The concentration of nitrite was determined by reading at 570 nm using ELISA reader.

### 2.6. Measurement of PGE_2_ Production

The RAW 264.7 cells were cultured in 24-well culture plates (5 × 10^5^ cells/mL). The cells were pretreated with various concentrations of KIOM-MA for 30 min and stimulated for 24 hours with LPS. The supernatant was used to determine PGE_2_ levels using a PGE_2_ Express EIA Kit (Cayman Chemical, Ann Arbor, MI, USA) in accordance with the manufacturer's instructions.

### 2.7. Enzyme-Linked Immunosorbent Assay (ELISA) for TNF-*α* and IL-6 Cytokine Detection

 Cells were seeded at a density of 5 × 10^5^ cells/mL in 24-well culture plates and pretreated with KIOM-MA for 30 min before LPS stimulation. ELISA plates (Nunc, Roskilde, Denmark) were coated overnight at 4°C with TNF-*α* or IL-6 antibody diluted in 0.1 M carbonate and then washed five times with phosphate-buffered saline (PBS) containing 0.05% Tween 20. The nonspecific protein-binding sites were blocked with assay diluent buffer (PBS containing 10% FBS, pH 7.0) for more than 1 hour. Promptly, samples and standards were added to each well. After 2 hours of incubation at RT or overnight at 4°C, the working detector solution (biotinylated TNF-*α* or IL-6 antibody and streptavidin-HRP reagent) was added and more incubated for 1 hour. Subsequently, substrate solution (tetramethylbenzidine) was added to the wells and incubated for 30 min in darkness before the reaction was stopped with stop solution (2 N H_3_PO_4_). The absorbance at 450 nm was measured. All subsequent steps were conducted at RT and all standards and samples were assayed in duplicate.

### 2.8. Cell Migration Assay

Macrophage migration assay was established using 24-well transwell unit with polycarbonate filters that have a diameter of 6.5 mm and a pore size of 8.0 *μ*m (Corning Costar, Cambridge, MA, USA) according to a reference previously reported with some modifications [17–19]. The cells (2 × 10^5^ cells) were loaded into top chamber containing 200 *μ*L of serum-free RPMI 1640 and pretreated with KIOM-MA. After LPS stimulation, the chambers were incubated for 48 hours at 37°C in a humidified 5% CO_2_ incubator. Cells that had migrated to lower surfaces of filter were fixed with 20% methanol for 30 min and stained with 0.2% crystal violet for 30 min. Unmigrated macrophages on the surface of upper membrane were replaced using cotton swab. Migrated macrophages were quantified by counting 5 random spots per each independent sample.

### 2.9. Gelatin Zymography Assay

 RAW 264.7 cells were seeded in 6-well culture plates (2 × 10^6^ cells/well). After the medium was changed with serum-free RPMI 1640 medium, the cells were pretreated with KIOM-MA for 30 min and then stimulated with LPS for 48 hours. After the collection of culture supernatant, cell debris in the culture supernatant was discarded by centrifugation. The same volume of culture supernatant was electrophoresed in an 8% SDS-PAGE containing 0.1% gelatin A (Sigma, St. Louis, MO, USA). Gels were washed twice with washing buffer (50 mM Tris-HCl, pH 7.5, 100 mM NaCl, and 2.5% Triton X-100) at RT for 30 min and then incubated in an incubation buffer (50 mM Tris-HCl, pH 7.5, 150 mM NaCl, 10 mM CaCl_2_. 0.02% NaN_3_, and 1 *μ*M ZnCl_2_) at 37°C for 24 hours, stained with 0.05% Coomassie brilliant blue, 10% isopropanol, and 10% acetic acid and destained with 10% isopropanol, 10% acetic acid. MMP-9 and MMP-2 were detected as a white clear zone in a dark blue field.

### 2.10. Western Blot Analysis

Protein expression was evaluated by Western blot analysis according to standard procedures. The cells were pretreated with KIOM-MA and stimulated with LPS for indicated periods at 37°C. After incubation, the cells were resuspended in protein lysis buffer (PRO-PERP, iNtRON, Sungnam, Republic of Korea) and cell debris was discarded by centrifugation. The protein concentration present in the supernatant was determined using the Bradford reagent (Bio-Rad Laboratories, Hercules, CA, USA) and equal amounts of protein were subjected to sodium dodecyl sulfate-polyacrylamide gel electrophoresis (SDS-PAGE). The membrane was blocked with 3% BSA in tris-buffered saline (150 mM NaCl, 20 mM Tris-HCl, and pH 7.4) with 0.05% Tween 20 (TBS-T) buffer. After blocking, the membrane was incubated with primary antibodies against iNOS, COX-2, NF-*κ*B p65, phospho-I*κ*B*α*, I*κ*B*α*, *β*-actin, TBP, phospho-ERK, ERK, phospho-p38, p38, phospho-JNK, and JNK (1 : 1000 diluted in TBS-T containing 3% BSA) for 18 hours at 4°C. The membrane was then washed with TBS-T and incubated with anti-mouse or anti-rabbit horseradish peroxidase (HRP)-conjugated immunoglobulin G secondary antibodies (1 : 10000 diluted in TBS-T containing 3% BSA) for 1 hour at RT. The specific proteins were detected using enhanced chemiluminescence (ECL) (Millipore, Billerica, MA, USA). The quantitation of protein was done using Davinch-chemi Chemiluminescence Imaging System CAS-400SM (Core Bio, Seoul, Republic of Korea).

### 2.11. Preparation of Cytosolic and Nuclear Extracts for NF-*κ*B and I*κ*B*α*

 The cells pretreated with KIOM-MA were stimulated by LPS. For the isolation of cytosolic fractions, the cells were washed twice in ice-cold PBS and incubated on ice for 10 min in lysis buffer (25 mM Tris-HCl (pH 7.4), 150 mM NaCl, 1 mM CaCl_2_, 1% Triton X-100, 1 mM PMSF, and 10 *μ*L/mL aprotinin), and the supernatant was collected after 10 min of centrifugation at 15,000 ×g at 4°C. To prepare the nuclear fractions, the cells were washed with 1 mL of ice-cold PBS, resuspended in 400 *μ*L of ice-cold hypotonic low-salt buffer (10 mM HEPES-KOH (pH 7.9), 10 mM KCl, 2 mM MgCl_2_, 0.1 mM EDTA, 1 mM DTT, and 0.5 mM PMSF), left on ice for 10 min, vortex-mixed and centrifuged for 30 sec at 15,000 g. The pellets were resuspended in 50 *μ*L of ice-cold high-salt buffer (50 mM HEPES-KOH (pH 7.9), 50 mM KCl, 1 mM DTT, 300 mM NaCl, 0.1 mM EDTA, 10% glycerol, and 0.5 mM PMSF), left on ice for 20 min, vortex-mixed, and centrifuged for 5 min at 15,000 ×g at 4°C to save the nuclear fraction. The cytosolic and nuclear extracts were used for the detection of NF-*κ*B p65 or I*κ*B*α* via Western blot analysis as described previously.

### 2.12. RNA Extraction and Reverse Transcription-Polymerase Chain Reaction (RT-PCR)

Total cellular RNA was isolated using the easy-BLUE RNA extraction kit (iNtRON, Sungnam, Republic of Korea) according to the procedure described by the manufacturer. The total RNA (2 *μ*g) was converted to cDNA using RevoScript RT PreMix (iNtRON, Sungnam, Republic of Korea). Then, cDNA was amplified by polymerase chain reaction using specific primers (Table 1). The following PCR conditions were used: TNF-*α*, IL-6, iNOS, COX-2, and *β*-actin, 35 cycles of denaturation at 94°C for 30 seconds, annealing at 65°C (TNF-*α*), 57°C (IL-6), 50°C (COX-2), 60°C (iNOS), and 57°C (*β*-actin) for 30 seconds, and extension at 72°C for 1 min [13, 20, 21].

### 2.13. Chromatographic Conditions

The HPLC-DAD system (Hitachi, Tokyo, Japan) consisted of a pump (L-2130), autosampler (L-2200), column oven (L-2350), and diode array UV/VIS detector (L-2455). The output signal of the detector was recorded using an EZchrom Elite software for Hitachi. For analysis of sample, an OptimaPak C18 (4.6 mm × 250 mm, 5 *μ*m, RS tech, Seoul, Republic of Korea) was used and the UV wavelength was 254 nm. The mobile phase was water and acetonitrile with a gradient elution (Table 2) at a flow rate of 1.0 mL/min and the column temperature was maintained at 40°C. Ten microliters of samples were injected to the column.

### 2.14. Preparation of Standard Solutions and Samples

 Each standard solution was prepared by dissolving each compound, namely, liquiritin, nodakenin, icariin, glycyrrhizin, decursin, and decursinol angelate in 100% methanol at the concentration of 100 *μ*g/mL. To prepare analytical samples, KIOM-MA powder was accurately weighed and dissolved with 100% H_2_O at a concentration of 40 mg/mL. Prior to analysis, sample preparation was filtered through a 0.45 *μ*m filter.

### 2.15. Statistical Analysis

The results are expressed as mean ± SD values for the number of experiments. Statistical significance was compared in each treated group with the negative control and determined by one-way ANOVA test. Each experiment was repeated at least three times to yield comparable results. Values with *P* < 0.05 and *P* < 0.01 were considered significant.

## 3. Results

### 3.1. Inhibitory Effect of KIOM-MA on NO and PGE_2_ Production

**Figure 1:**
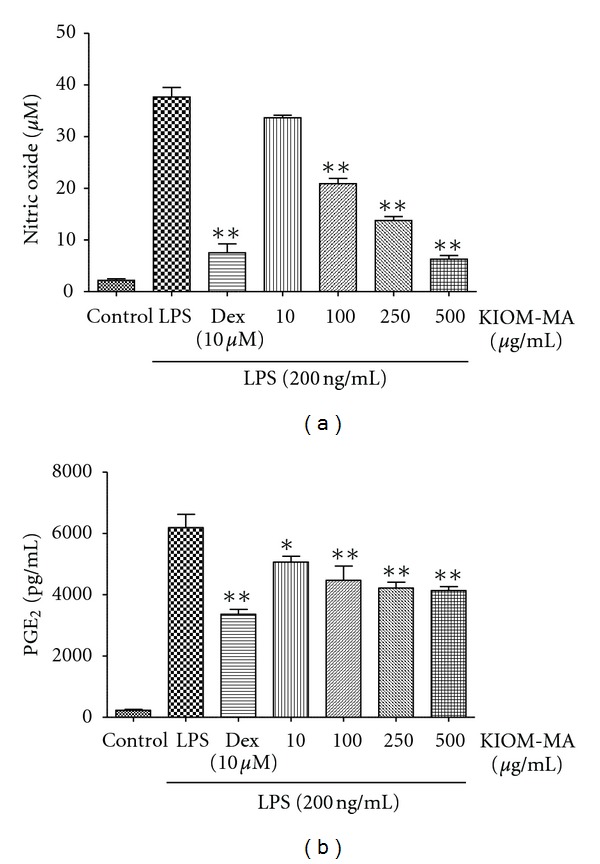
Effect of KIOM-MA on LPS-induced (a) NO and (b) PGE_2_ production in RAW 264.7 cells. RAW 264.7 cells were pretreated with KIOM-MA for 30 min before being incubated with LPS for 24 hours. The culture supernatant was analyzed for nitrite or PGE_2_ production. As a control, the cells were incubated with vehicle alone. Data shows mean ± SD values of duplicate determinations from three independent experiments. *P < 0.05 and **P < 0.01 were calculated from comparing with LPS-stimulation value.

To investigate the effect of KIOM-MA on inflammation, we checked the levels of NO and PGE_2_ secreted upon LPS stimulation in macrophage cells. KIOM-MA at four concentrations (10, 100, 250, and 500 *μ*g/mL) was treated to the cells before LPS stimulation and measured NO and PGE_2_ production. Since dexamethasone is widely used for anti-inflammatory agent and contains a stronger efficacy compared with hydrocortisone, we used dexamethasone (10 *μ*M) as a positive control. In Figure 1(a), KIOM-MA strongly repressed NO production in a dose-dependent manner. Especially, KIOM-MA at 250 and 500 *μ*g/mL inhibited NO production more than 64% and 83% compared with LPS alone, respectively. Moreover, KIOM-MA inhibited PGE_2_ production and showed statistical significance at concentrations of 250 and 500 *μ*g/mL (Figure 1(b)). These results indicate that KIOM-MA effectively suppresses the secretion of nitrite and PGE_2_ from LPS-stimulated RAW 264.7 macrophage cells.

### 3.2. KIOM-MA Represses Proinflammatory Cytokine Expression in RAW 264.7 Cells

**Figure 2:**
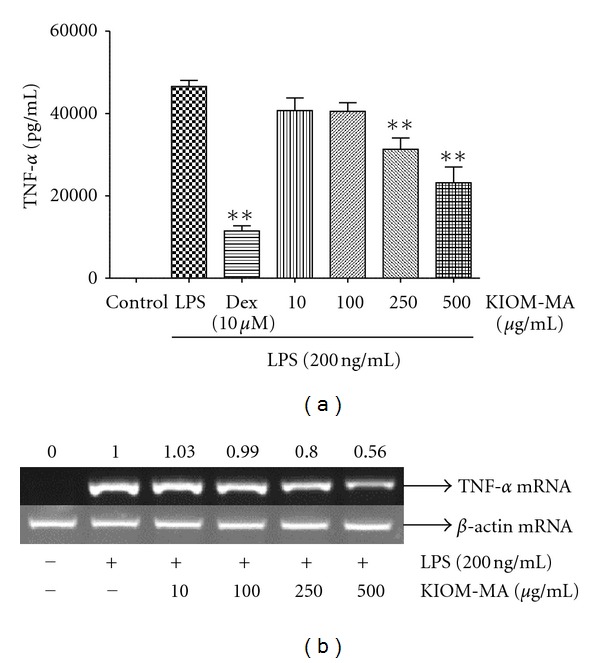
Effect of KIOM-MA on TNF-*α* (a) cytokine production and (b) mRNA expression upon LPS stimulation. RAW 264.7 cells were pretreated with KIOM-MA for 30 min before being incubated with LPS for (a) 24 hours and (b) 6 hours, respectively. Production of TNF-*α* cytokine was measured by ELISA and mRNA level was analyzed by RT-PCR. Data shows mean ± SD values of duplicate determinations from three independent experiments. *P < 0.05 and **P < 0.01 were calculated from comparing with LPS-stimulation value. The level of *β*-actin mRNA was compared in parallel to confirm the equivalency of the cDNA preparation. The RNA value in the cells stimulated by LPS without KIOM-MA treatment was set to 1.00 and the effect by treatment was quantitated using i-MAX Gel Image Analysis System (Core Bio, Seoul, Republic of Korea). The experiment was repeated three times.

**Figure 3:**
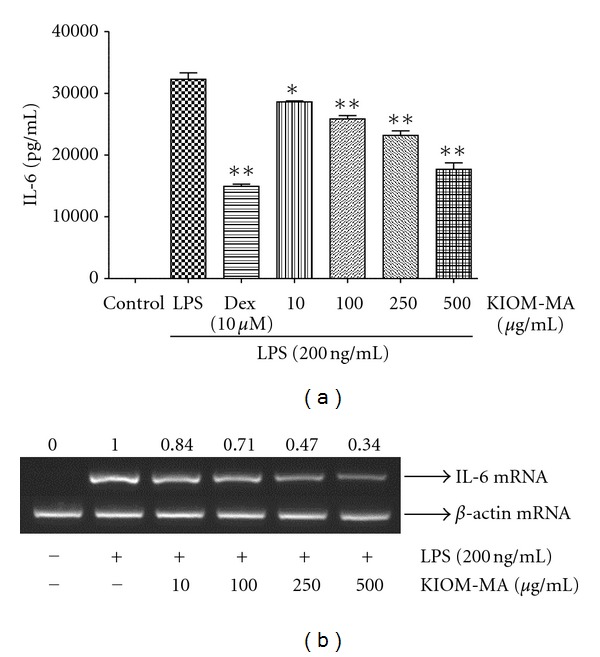
Effect of KIOM-MA on LPS-induced IL-6 (a) cytokine production and (b) mRNA expression. RAW 264.7 cells were pretreated with KIOM-MA for 30 min before being incubated with LPS for (a) 24 hours and (b) 6 hours, respectively. Production of IL-6 cytokine was measured by ELISA and mRNA level was analyzed by RT-PCR. Data shows mean ± SD values of duplicate determinations from three independent experiments. *P < 0.05 and **P < 0.01 were calculated from comparing with LPS-stimulation value. The level of *β*-actin mRNA was compared in parallel to confirm the equivalency of the cDNA preparation. The RNA value in the cells stimulated by LPS without KIOM-MA treatment was set to 1.00 and the effect by treatment was quantitated using i-MAX Gel Image Analysis System (Core Bio, Seoul, Republic of Korea). The experiment was repeated three times.

To investigate the effect of KIOM-MA on TNF-*α* and IL-6 produced by LPS stimulation, ELISA and RT-PCR analyses were conducted. The cells pretreated with KIOM-MA at the different concentrations got stimulated with LPS and the levels of TNF-*α* and IL-6 were measured. As shown in Figure 2, KIOM-MA effectively inhibited both protein and mRNA expression of TNF-*α* at the concentration of 500 *μ*g/mL and the effect was increased in a dose-dependent manner. When we also checked the effect of KIOM-MA on the production of other cytokine IL-6, consistent with TNF-*α* result, KIOM-MA inhibited the production of IL-6 cytokine and mRNA expression dose dependently, as presented in Figure 3. Specially, KIOM-MA showed a strong suppressive effect on IL-6 production more than 50% at concentrations of 250 and 500 *μ*g/mL.

### 3.3. KIOM-MA Inhibits LPS-Induced iNOS and COX-2 Expression

**Figure 4:**
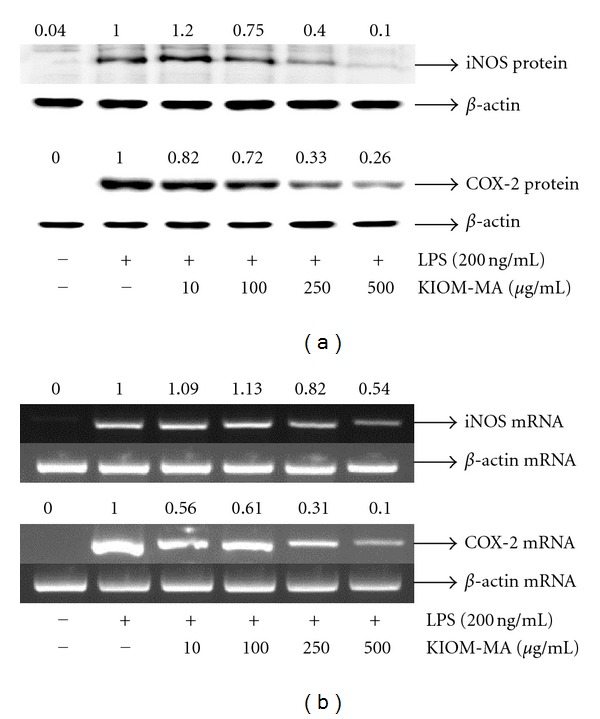
Effect of KIOM-MA on the expression of iNOS or COX-2 (a) protein and (b) mRNA in LPS-stimulated RAW 264.7 cells. RAW 264.7 cells were pretreated with KIOM-MA for 30 min before being incubated with LPS for 24 hours. Equal amounts of protein were separated by SDS-PAGE and immunoblotted with iNOS and COX-2 antibodies. Equal loading of protein was confirmed by *β*-actin. iNOS and COX-2 mRNAs were analyzed by RT-PCR in RAW 264.7 cells. The *β*-actin mRNA was used to confirm the equivalency of the cDNA preparation. The protein or mRNA value in the LPS-stimulated cells without KIOM-MA treatment was set to 1.00 and the effect by treatment was quantitated. The experiment was repeated three times.

We investigated the inhibitory effect of KIOM-MA on iNOS and COX-2 expression, which are synthetase of NO and PGE_2_, respectively. In Figure 4(a), KIOM-MA highly decreased the level of iNOS and COX-2 protein at concentrations of 250 and 500 *μ*g/mL. Also, KIOM-MA significantly repressed iNOS and COX-2 mRNA expression in a dose-dependent fashion without affecting the level of *β*-actin used as a control (Figure 4(b)). These results suggest that KIOM-MA suppresses both iNOS and COX-2 expression in the transcriptional level in addition to protein level, resulting in the reduced expression of NO and PGE_2_.

### 3.4. KIOM-MA Suppresses LPS-Induced Migration and Gelatinase Activity of MMP-9 in Macrophage

**Figure 5:**
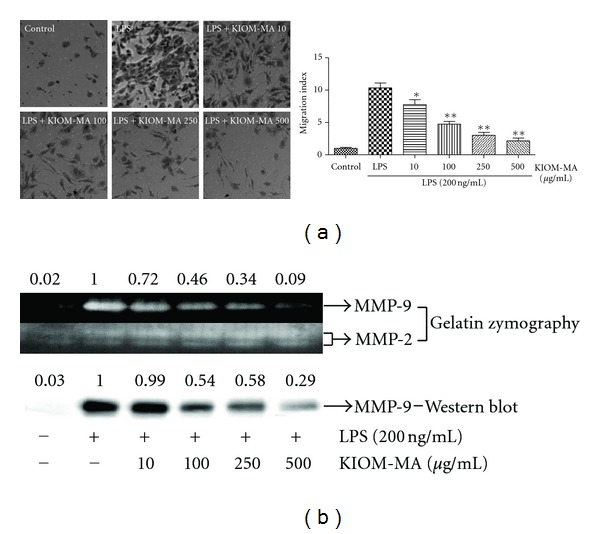
Effect of KIOM-MA on LPS-induced (a) RAW 264.7 cell migration, (b) activity and expression of MMP-9. Cells were pretreated with indicated concentrations of KIOM-MA for 30 min before LPS stimulation. After incubation for 48 hours, (a) cells on lower filter surfaces were fixed and stained with 20% methanol/0.2% crystal violet (v/v). Migrated cells were determined under microscope by counting cells in 5 random fields. Data shows mean ± SD values from three independent experiments. *P < 0.05 and **P < 0.01 were calculated from comparing with LPS-stimulation value. (b) The culture supernatant of RAW 264.7 cell was prepared and used for gelatin zymography and Western blot analysis. The experiment was repeated three times independently.

On the base of inhibitory effects of KIOM-MA on the expression of COX-2 and PGE_2_, we investigated whether KIOM-MA could inhibit the migration and MMP-9 activity and/or MMP-9 expression in LPS-stimulated macrophage cells. As shown in Figure 5(a), LPS treatment increased the migration of RAW 264.7 cells compared with untreated control cells in transwell migration assay. When cells were pretreated with various concentrations of KIOM-MA, cell migration was significantly inhibited, in a dose-dependent manner. Specially, KIOM-MA at 500 *μ*g/mL inhibited migration more than 80% compared with LPS-treated control group. Because KIOM-MA represses the migration of macrophage, we also investigated the activity and expression of MMP-9, which is concerned in cell migration. When we checked the effect of KIOM-MA on MMP-9 activity by gelatin zymography and MMP-9 expression by Western blot, KIOM-MA significantly inhibited gelatinase activity as well as expression of MMP-9 in a dose-dependent manner (Figure 5(b)) and significantly correlated with migration capacity of LPS-stimulated macrophages.

### 3.5. Inhibitory Effect of KIOM-MA on Nuclear Translocation of NF-*κ*B and I*κ*B*α* Degradation by LPS Stimulation

**Figure 6:**
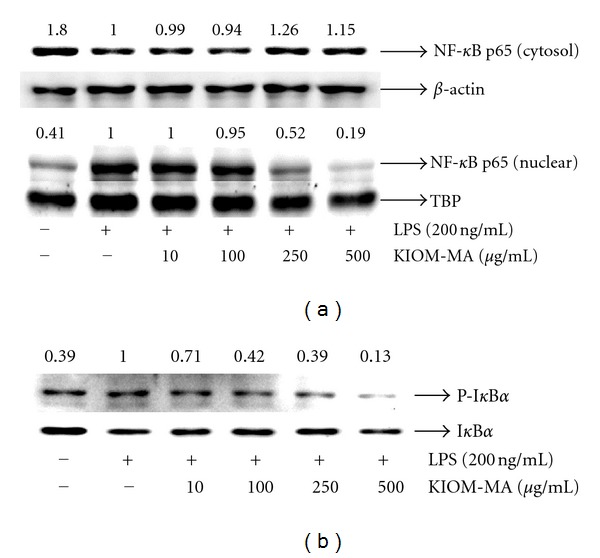
Effect of KIOM-MA on (a) translocation of the NF-*κ*B (p65) subunit into the nucleus and (b) release of I*κ*B*α* into the cytosol upon LPS stimulation. The cells were treated with LPS alone or with LPS and KIOM-MA for 1 hour. The level of I*κ*B*α* protein in the cytosol and NF-*κ*B (p65) protein present in the cytosol and nucleus was determined by the Western blot analysis using anti-I*κ*B*α* or anti-NF-*κ*B p65 antibody, as described in Section 2. *β*-actin and TBP were used for cytosolic and nuclear control protein, respectively. The protein value in the cells stimulated by LPS without KIOM-MA treatment was set to 1.00 and the effect by treatment was quantitated. The experiment was repeated three times independently.

 Expressions of iNOS and COX-2 genes are induced by NF-*κ*B activation. Upon activation, p65 protein of NF-*κ*B is translocated from cytosol to nucleus. Therefore, we checked the level of p65 protein in the cytoplasm and nucleus using the Western blot analysis. As shown in Figure 6(a), the nuclear translocation of p65 by LPS stimulation was strongly inhibited in the presence of KIOM-MA at a concentration of 250 *μ*g/mL, and the inhibitory effect was increased in a dose-dependent manner. We also examined whether the inhibition of p65 by KIOM-MA affects the levels of I*κ*B*α* and phosphorylated I*κ*B*α* in the cytoplasm. As a result, Figure 6(b) shows that KIOM-MA significantly repressed I*κ*B*α* phosphorylation dose dependently, implying that KIOM-MA prevents I*κ*B*α* degradation and NF-*κ*B activation.

### 3.6. Effect of KIOM-MA on the Phosphorylation of MAPKs in LPS-Stimulated RAW 264.7 Cells

**Figure 7:**
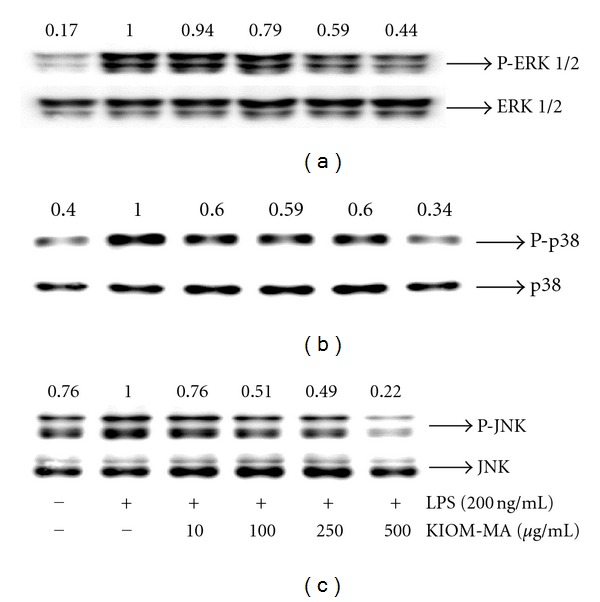
Effect of KIOM-MA on the phosphorylation of MAPKs in LPS-stimulated RAW 264.7 cells: (a) ERK1/2 MAPK, (b) p38 MAPK, and (c) JNK MAPK. RAW 264.7 cells were treated with KIOM-MA for 30 min before being incubated with LPS for 30 min. Cell lysates were analyzed by the Western blot analysis using specific antibodies. The protein value in the cells stimulated by LPS without KIOM-MA treatment was set to 1.00 and the effect by treatment was quantitated. The experiment was repeated three times independently.

We investigated whether MAPKs are involved in the inhibition of NF-*κ*B activation by KIOM-MA. To address that, we examined the effect of KIOM-MA on the phosphorylation of three MAPKs in LPS-stimulated RAW 264.7 cells. KIOM-MA reduced the levels of all MAPK phosphorylation (Figure 7). Especially, KIOM-MA significantly inhibited the activities of p38 and JNK MAPKs, in a dose-dependent manner (Figures 7(b) and 7(c)).

### 3.7. HPLC Analysis of KIOM-MA

**Figure 8:**
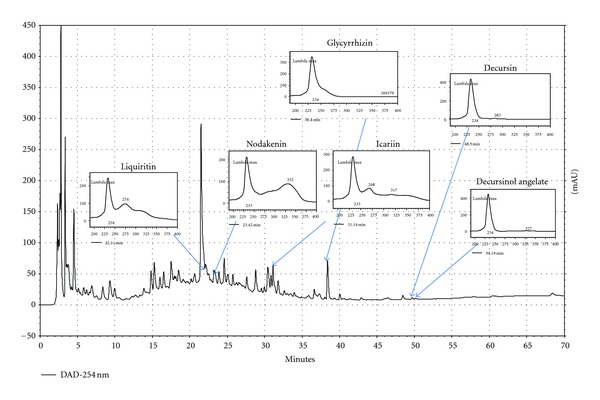
The representative chromatogram and UV spectrum of KIOM-MA at 254 nm; liquiritin, nodakenin, icariin, glycyrrhizin, decursin, and decursinol angelate.

The main components profile of KIOM-MA was analyzed via HPLC. The representative chromatogram is shown in Figure 8. The identification of constituent of KIOM-MA was based on the retention times and UV spectrum in comparison with authentic standards at a wavelength of 254 nm. Peak purity check and identification were conducted via a 190–400 nm UV scan through a diode array detector. Six components (liquiritin, 22.11 min; nodakenin, 23.42 min; icariin, 31.14 min; glycyrrhizin, 38.40 min; decursin, 48.50 min; decursinol angelate, 50.19 min) were identified in KIOM-MA.

## 4. Discussion

Many previous studies on natural herb and herbal medicines have conducted to find the potential natural anti-inflammatory products through *in vitro* and *in vivo* systems. KIOM-MA is a novel herbal medicine composed of several herbs, which are traditionally used for the treatment of inflammatory and allergic diseases. Since ancient times, Glycyrrhizae Radix has been used for the treatment of dermatitis and eczema and Sophorae Radix and Arctii Fructus have been prescribed for hepatitis, jaundice and laryngopharyngitis, cough, and sputum. Recently, our group reported that KIOM-MA contains the inhibitory activities on atopic dermatitis and asthma [15, 16].

**Figure 9:**
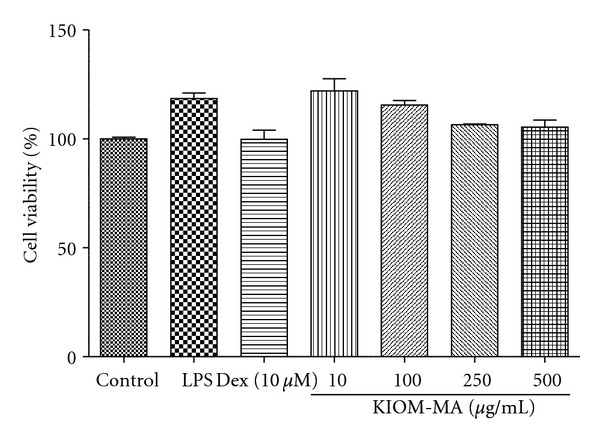
The cytotoxicity of KIOM-MA on RAW 264.7 cells at different doses was evaluated by an MTT assay.

In this study, we have demonstrated the anti-inflammatory activity of KIOM-MA in LPS-stimulated RAW 264.7 macrophage cells. Because the overproduction of NO is closely related with many inflammatory diseases [22, 23] and PGE_2_ is also a major indicator of inflammation, we first examined the effect of KIOM-MA on the secretion of NO and PGE_2_. As a result, KIOM-MA effectively inhibited LPS-induced NO production at high concentrations. When we checked the cytotoxicity of KIOM-MA using an MTT assay, KIOM-MA did not affect the viability of RAW 264.7 cells, up to a concentration of 500 *μ*g/mL (Figure 9). We further explored whether the reduced production of NO and PGE_2_ by KIOM-MA is originated from the inhibition of their synthesis. KIOM-MA significantly suppressed the expression of both iNOS and COX-2 in a dose-dependent manner. These results suggest that KIOM-MA contains a strong inhibitory activity on a proinflammatory mediator production.

NF-*κ*B is a main regulatory transcription factor involved in cellular responses to various stimuli such as stress, cytokines, free radicals, ultraviolet irradiation, oxidized LDL, and bacterial or viral antigens [24–28]. In addition, NF-*κ*B plays a pivotal role in the adjustment of cell survival and the expression of proinflammatory cytokines [29–33]. When we investigated whether KIOM-MA inhibits the activation of NF-*κ*B, we found that nuclear translocation of p65 upon LPS stimulation was blocked by KIOM-MA in a dose-dependent manner through the inhibition of I*κ*B*α* degradation. These findings are consistent with other reports, demonstrating that NF-*κ*B response elements are present on the promoter of the iNOS, COX-2, and the inflammatory cytokine genes [34–37].

Because activated MAPKs take an important role in LPS-induced iNOS expression signaling pathway in mammalian cells [38], we also investigated the effect of KIOM-MA on the MAPKs phosphorylation in RAW 264.7 cells. KIOM-MA significantly decreased the levels of phosphorylation of p38 and JNK MAPKs in a dose-dependent manner. These results suggest that the inhibitory effect of KIOM-MA on the phosphorylation of MAPKs was directly involved in the reduced production of proinflammatory mediators in RAW 264.7 cells.

MMP-9 is controlled by the inflammatory mediator NO and PGE_2_ [5, 39]. Also, MMP-9 activity and expression were related to the ability of cell migration. In other words, inhibitory activity on NO and PGE_2_ production was directly or indirectly related with macrophage migration. Therefore, we examined the effect of KIOM-MA on cell migration and MMP-9 activity, expression upon LPS stimulation. KIOM-MA dose dependently repressed the migration of macrophages and gelatinase activity, expression of MMP-9 and showed a statistical significance.

As presented in Figure 8, HPLC analysis identified six main components (liquiritin, nodakenin, icariin, glycyrrhizin, decursin, and decursinol angelate) in KIOM-MA. A previous study reported that liquiritin exerts an anti-inflammatory effect by inhibiting NO and PGE_2_ production in LPS-stimulated RAW 264.7 cells [40]. Additionally, it was demonstrated that nodakenin suppresses NO production in RAW 264.7 cells [41]. Another recent study has revealed that icariin attenuates LPS-induced inflammation via the inhibition of the CD14/TLR4 signaling pathway in human monocytes [42] and represses LPS-induced acute inflammatory responses through PI3 K/Akt and NF-*κ*B signaling [43]. Also, a previouse study showed that glycyrrhizin suppresses LPS sensor Toll-like receptor 4/MD-2 complex signaling [44]. In addition, according to a previous study, decursin contains the inhibitory activity on inflammatory mediators by blocking NF-*κ*B activation in macrophages [45]. These facts suggest that the anti-inflammatory activity of KIOM-MA might be related to active components in KIOM-MA, such as liquiritin, nodakenin, icariin, glycyrrhizin, decursin, and decursinol angelate.

In conclusion, KIOM-MA exerts significant inhibitory effects on the production of NO, PGE_2_, and inflammatory cytokines such as TNF-*α*, IL-6 and iNOS, and COX-2 expressions in LPS-stimulated RAW 264.7 cells and this anti-inflammatory effect was mediated by the suppression of NF-*κ*B activation through I*κ*B*α* stabilization and the blockage of MAPK phosphorylation. Additionally, KIOM-MA contains a strong suppressive effect on LPS-induced macrophage migration, MMP-9 gelatinase activity and expression. These results support that KIOM-MA could be developed as a new anti-inflammatory herbal medicine without cytotoxicity after further *in vivo* studies.

## Figures and Tables

**Table 1:** Primers used for RT-PCR analysis.

Target gene	Primer sequence
TNF-*α*	F: 5′-AGCCCACGTCGTAGCAAACCACCAA-3′
	R: 5′-AACACCCATTCCCTTCACAGAGCAAT-3′

IL-6	F: 5′-CATGTTCTCTGGGAAATCGTGG-3′
	R: 5′-AACGCACTAGGTTTGCCGAGTA-3′

iNOS	F: 5′-CCTCCTCCACCCTACCAAGT-3′
	R: 5′-CACCCAAAGTGCTTCAGTCA-3′

COX-2	F: 5′-ACTCACTCAGTTTGTTGAGTCATTC-3′
	R: 5′-TTTGATTAGTACTGTAGGGTTAATG-3′

*β*-actin	F: 5′-TGGAATCCTGTGGCATCCATGAAA-3′
	R: 5′-TAAAACGCAGCTCAGTAACAGTCCG-3′

F: forward; R: reverse.

**Table 2:** Mobile phase condition of chromatographic separation.

Time (min)	Water (in 2% acetic acid)	Acetonitrile (in 2% acetic acid)	Flow rate (mL/min)
0	95	5	1
5	95	5	1
60	20	80	1
70	20	80	1
75	95	5	1
90	95	5	1
